# First assessment of underwater sound levels in the Northern Adriatic Sea at the basin scale

**DOI:** 10.1038/s41597-023-02033-1

**Published:** 2023-03-15

**Authors:** Antonio Petrizzo, Andrea Barbanti, Giulia Barfucci, Mauro Bastianini, Ilaria Biagiotti, Sofia Bosi, Michele Centurelli, Robert Chavanne, Antonio Codarin, Ilaria Costantini, Marinela Cukrov Car, Vlado Dadić, Francesco M. Falcieri, Raffaela Falkner, Giulio Farella, Mario Felli, Christian Ferrarin, Thomas Folegot, Roger Gallou, Daphnie Galvez, Michol Ghezzo, Aleksandra Kruss, Iole Leonori, Stefano Menegon, Hrvoje Mihanović, Stipe Muslim, Alice Pari, Sauro Pari, Marta Picciulin, Grgur Pleslić, Marko Radulović, Nikolina Rako-Gospić, Davide Sabbatini, Giulia Soldano, Jarosław Tęgowski, Tihana Vučur-Blazinić, Predrag Vukadin, Jakub Zdroik, Fantina Madricardo

**Affiliations:** 1 CNR-National Research Council, ISMAR - Institute of Marine Sciences in Venice, Castello 2737/f, 30122 Venice, Italy; 2 ARPA FVG — Regional Environmental Protection Agency of Friuli Venezia Giulia, via Cairoli 14, 33057 Palmanova, Udine Italy; 3 CNR-National Research Council, IRBIM -Institute of Marine Biological Resources and Biotechnologies, SS Ancona, Largo Fiera della Pesca, 1 - 60125 Ancona, Italy; 4 Quiet Oceans, Bâtiment Cap Ocean, Technopôle Brest-Iroise, 525 avenue Alexis de Rochon, 29280 Plouzané, France; 5 Blue World Institute of Marine Research and Conservation, Kaštel 24, 51551 Veli Lošinj, Croatia; 6 Institute of Oceanography and Fisheries (IOR), Šetalište I. Meštrovića 63, 21000 Split, Croatia; 7 CNR-National Research Council, INM - Institute of Marine Engineering, via di Vallerano 139, 00128 Roma, Italy; 8 NORBIT Poland, ul Niepodleglosci 813-815, lok.24, 81-810 Sopot, Poland; 9 Fondazione Cetacea Onlus, Viale Torino 7A, 47838 Riccione, RN Italy; 10 Institute of Oceanography, University of Gdańsk, Av. Marszałka Piłsudskiego 46, 81-378 Gdynia, Poland

**Keywords:** Environmental sciences, Physical oceanography

## Abstract

The protection of marine habitats from human-generated underwater noise is an emerging challenge. Baseline information on sound levels, however, is poorly available, especially in the Mediterranean Sea. To bridge this knowledge gap, the SOUNDSCAPE project ran a basin-scale, cross-national, long-term underwater monitoring in the Northern Adriatic Sea. A network of nine monitoring stations, characterized by different natural conditions and anthropogenic pressures, ensured acoustic data collection from March 2020 to June 2021, including the full lockdown period related to the COVID-19 pandemic. Calibrated stationary recorders featured with an omnidirectional Neptune Sonar D60 Hydrophone recorded continuously 24 h a day (48 kHz sampling rate, 16 bit resolution). Data were analysed to Sound Pressure Levels (SPLs) with a specially developed and validated processing app. Here, we release the dataset composed of 20 and 60 seconds averaged SPLs (one-third octave, base 10) output files and a Python script to postprocess them. This dataset represents a benchmark for scientists and policymakers addressing the risk of noise impacts on marine fauna in the Mediterranean Sea and worldwide.

## Background & Summary

Underwater ambient sound levels are a critical component for the health of the marine ecosystems. Marine organisms are evolved to get relevant information by listening to the soundscape, whose acoustic signature reveals the occurrence of natural events and vocal species^1,2^. In this context, the input of underwater noise induced by human activities has been linked to detrimental effects on marine fauna^3–7^. As a result, the anthropogenic underwater noise has been recognised as a pollutant of international concern and has been addressed by international agreements^8^. The U.S. National Oceanic and Atmospheric Administration’s Ocean Noise Strategy (ONS), for example, focuses on the evaluation and management of the human-generated noise and its effect on marine species, supporting the goals of the U.S. National Ocean Policy^9^. Further, the European Union’s Marine Strategy Framework Directive (MSFD) requires the EU member states to monitor and mitigate noise pollution to reach a “Good Environmental Status” of the marine environment. Setting up monitoring cross-border programmes aiming to evaluate the underwater sound levels at sub-regional scale is recommended in the MSFD context (EU Directive 2008/56/EC).

Global efforts on monitoring underwater sound levels resulted in long-term projects dedicated to target areas^10^, including, among others, the US Outer Continental Shelf and the US coastal waters (ADEON, NOAA CetSound Project, respectively), the British Columbia, the Vancouver Port waters, the Canadian Atlantic coast waters and the Gulf of St. Lawrence (ECHO program^11^, ESRF and SeaWays projects, respectively). Underwater soundscapes have been investigated also in Australia^12,13^, Eastern, Southern and South East Asia^14^ and South Africa^15^ waters, as well as in Artic^16^ and Antartic^17^ waters.

Continuous sound monitoring EU projects have been established in the Northeast Atlantic (JOMOPANS and JONAS), in the Baltic Sea (BIAS) and in waters between Scotland and Ireland (COMPASS)^18–21^. In contrast, no extensive research on the underwater sound continuous levels has been developed in the Mediterranean Sea so far. Pilot monitoring studies have been run in the context of the EU QUIETMED project^22^ together with few other local studies^23–25^, including some done in the Adriatic Sea^26–29^. The EU Interreg Italy-Croatia project SOUNDSCAPE (Soundscapes in the North Adriatic Sea and their impact on marine biological resources) has been therefore established to implement a shared monitoring network for a coordinated transnational assessment of the underwater ambient sound in the North Adriatic Sea (NAS). The Adriatic has been recognized as one of the important sub-regions of the Mediterranean Sea by the MSFD; the NAS is its shallowest, northernmost part. Most of NAS is considered to be an Ecologically and Biologically Significant Area (EBSA, Convention on Biological Diversity), as well as hosting several marine and coastal Natura 2000 sites, and protected areas^30^. Whilst having a very vulnerable biodiversity^31^, NAS is highly impacted by increasing maritime traffic, tourism and resource exploitation^32^. As a result, NAS biota is currently under the combined pressure of the anthropogenic impact^33^ and climate change^34,35^.

**Fig. 1:**
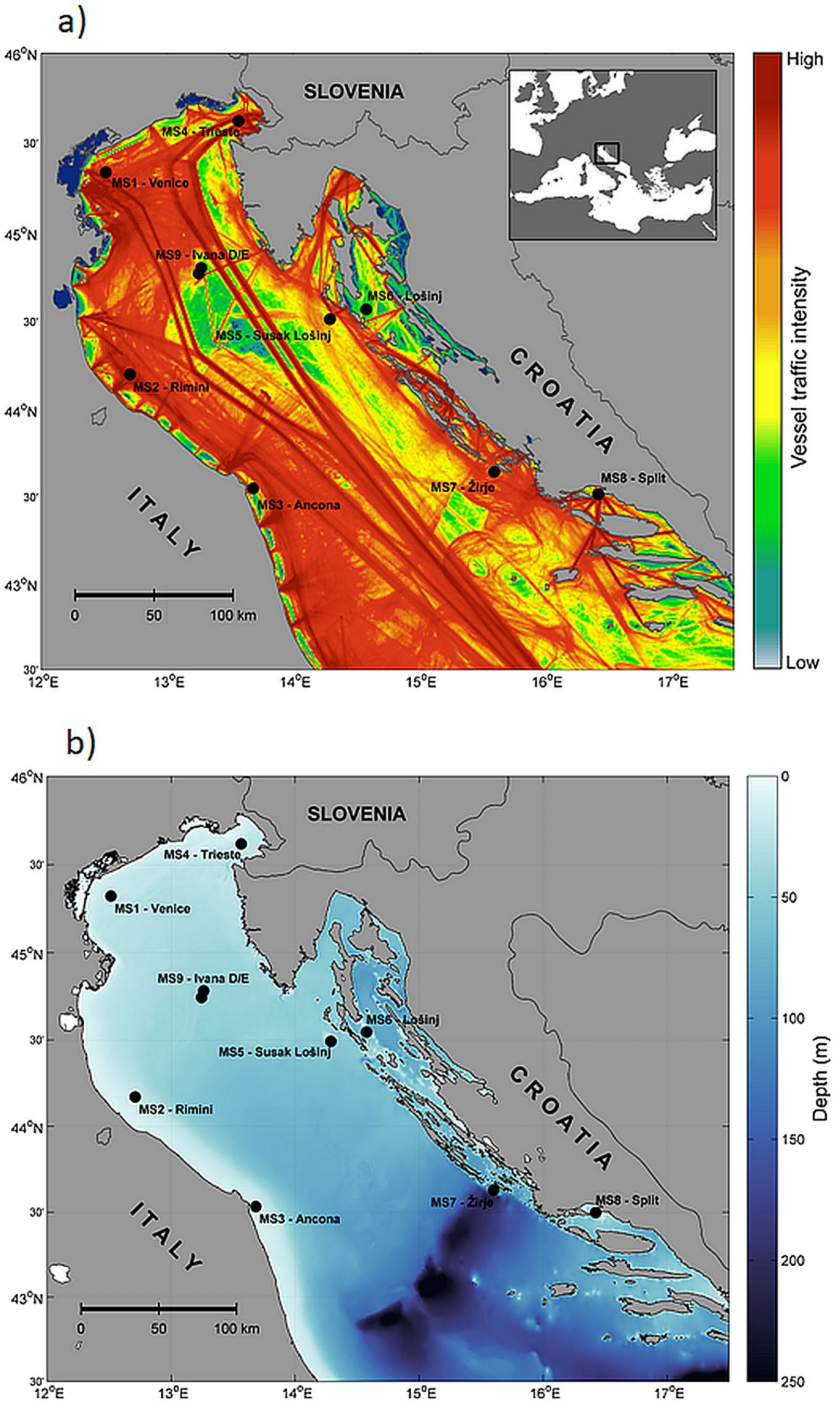
Soundscape recording stations in the Northern Adriatic Sea; vessel traffic (**a**) and bathymetry (**b**) are highlighted. Vessel traffic is represented as total number of vessel passages in 2020, obtained from EMODnet Human Activities, Vessel Density Map. (revision date 2022-03-21).

The SOUNDSCAPE dataset presented in this paper contains 20 and 60 seconds averaged sound pressure levels (SPLs) collected at nine monitoring stations, from the Gulf of Trieste till about the Middle Adriatic Pit (Fig. 1; Table 1), from March 2020 to June 2021. This dataset is essential for establishing baselines that document acoustic conditions over time on the regional scale and represents the first dataset of this kind in the Mediterranean Sea. The collected data are crucial to assess the ecosystem health, to evaluate the consequences of new possible marine development and to promote a knowledge-based management of the marine resources. Additionally, the SOUNDSCAPE dataset includes the most restrictive COVID-19-induced lockdown phase (March–April 2020), providing a unique benchmark for spatial and temporal comparative analysis.

**Table 1:** Coordinates, bottom depth and sediment types of the recording locations.

Monitoring station reference	Position		Water depth (m)	Sediment type
	Longitude (E)	Latitude (N)		
MS1 – Venice (IT)	12°30.883′	45°19.383′	17	sand
MS2 – Rimini (IT)	12°42.656′	44°10.254′	18	sand
MS3 – Ancona (IT)	13°40.932′	43°31.954′	15	sand
MS4 – Trieste (IT)	13°33.917′	45°37.095′	25	sandy mud
MS5 – Susak Lošinj(HR)	14°17.293′	44°29.545′	40	rocks/sand
MS6 - Lošinj (HR)	14°34.510′	44°32.747′	37	sand
MS7 – Žirje(HR)	15°36.020′	43°37.788′	46	gravelly sand
MS8 – Split (HR)	16°25.336′	43°29.895′	40	slightly sandy mud
*MS9 – Ivana D (HR)	13°15.720′	44°46.953′	42	terrigenous sand
*MS9 – Ivana E (HR) Since Dec. 2020	13°14.674′	44°44.687′	42	terrigenous sand

*MS9 position was changed because the Ivana D gas production platform collapsed in December 2020 and the whole area was closed and access restricted; the two positions are only few miles apart and the differences in the data collected are regarded not relevant.

## Methods

**Fig. 2:**
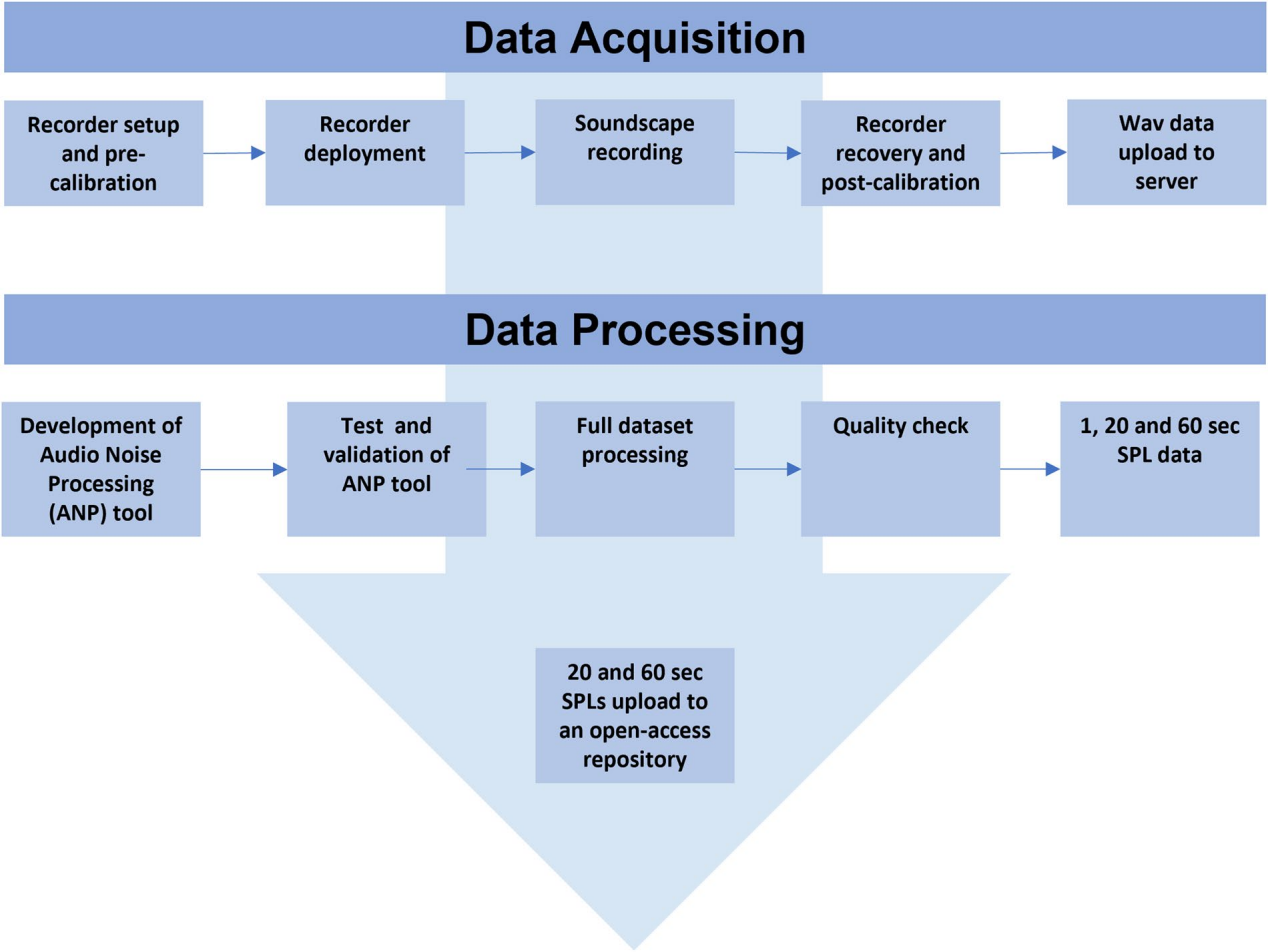
Workflow of the acquisition and processing of underwater noise data to obtain SPL_20,60_ dataset.

The workflow shown in Fig. 2 summarizes the steps undertaken to obtain the SPL datasets from the underwater noise raw data collected in the field. The workflow entails two main blocks: (i) “Data Acquisition” describes the process of sound recording and wav files uploading on the SOUNDSCAPE-dedicated server to store the data; (ii) “Data Processing” shows the steps that lead to the processing of wav data to calculate Sound Pressure Level data.

The applied procedures are in accord to guidelines developed by other international projects or agreements^10,36^. Used terminology followed ISO 18405^37^, IEC 61260-1:2014^38^ and JOMOPANS^39^ Terminology Standards and it is summarized in Table 2.

**Table 2:** Used acoustic terminology.

sound	alteration in pressure, stress or material displacement propagated via the action of elastic stresses in an elastic medium and that involves local compression and expansion of the medium^37^
signal	specified time-varying electric current, voltage, sound pressure, sound particle displacement, or other field quantity of interest^37^
self-noise	fluctuations in output of a receiver system caused by the combination of (i) acoustic self-noise, caused by the deployment, operation, or recovery of a specified receiver, and its associated platform^37^, and (ii) non-acoustic self-noise, such as electrical noise in the hydrophone and receiver electronics^39^
ambient noise	all sound except sound associated with a specified signal and except self-noise^37^
ambient sound	sound that is present in the absence of sound from a specified activity^37^
soundscape	characterization of the ambient sound in terms of its spatial, temporal and frequency attributes, and the types of sources contributing to the sound field^37^
sound pressure; p(t)	the difference between instantaneous total pressure and pressure that would exist in the absence of sound^37^
reference pressure; p(0)	1 µPa in underwater acoustics
RMS sound pressure; p(rms)	The square root of the mean square pressure; mean square pressure is the time integral of squared sound pressure (p(t)) over a specific time interval divided by the duration of the time interval^37^
sound pressure level (SPL) (mean-square sound pressure level)	20 log10 [p(rms)/p(0)] dB^37^
octave	logarithmic frequency interval between frequencies f1 and f2 when f2/f1 = 2^39^
decade	logarithmic frequency interval between frequencies f1 and f2 when f2/f1 = 10^39^
one-third octave (base 10) or decidecade band	one tenth of a decade^38^
percentile	a statistical measure indicating the value below which a given percentage of observations in a group of observations fall^39^
temporal observation window	interval of time within which a statistic of the sound pressure is calculated or estimated^39^
temporal analysis window	interval of time during which statistics are calculated over multiple temporal observation windows^39^

### Hardware components and calibration

The acoustic recordings were made by using autonomous passive underwater acoustic recorders (APUARs; Sono.Vault by Develogic Subsea Systems GmbH, Hamburg, Germany). Each recorder was featured with an omnidirectional broadband Neptune Sonar D60 Hydrophone characterized by a sensitivity around −192.7 dB re 1V/µPa (flat frequency response: 10 Hz – 20 kHz ± 3 dB). The processing chain includes a high-pass filter (cut-off frequency in the range of 3–10 Hz), a preamplifier and a 16 bit analogue to digital converter (ADC). The 16-Bit ADC has a high frequency reject filter with 500 kHz and it is otherwise limited by the input amplifier which has a bandwidth of approximately 100 kHz. The ADC is the last component in the processing chain. The data that is stored comes directly from the ADC.

The waterproof pressure resistant housing contained a programmable recorder with variable gain, a battery set consisting of lithium D-Cells and up to 1TB-SD memory cards.

Hydrophone calibration was achieved by the manufacturer using a calibrated reference projector; the reference projector was calibrated as well using a free-field three-transducer reciprocity calibration. Both procedures are compliant with the IEC 60565-1:2020 international standard^40^. The recorders were set to record continuously at a sampling rate of 48 kHz, providing a recording bandwidth of approximately 22 kHz with 16-bit resolution.

Additional information and hydrophone sensitivity curves in the pertinent frequency range are available in the SOUNDSCAPE Deliverables 3.2.1^41^ and 3.6.3^42^.

### Acoustic data acquisition

**Fig. 3:**
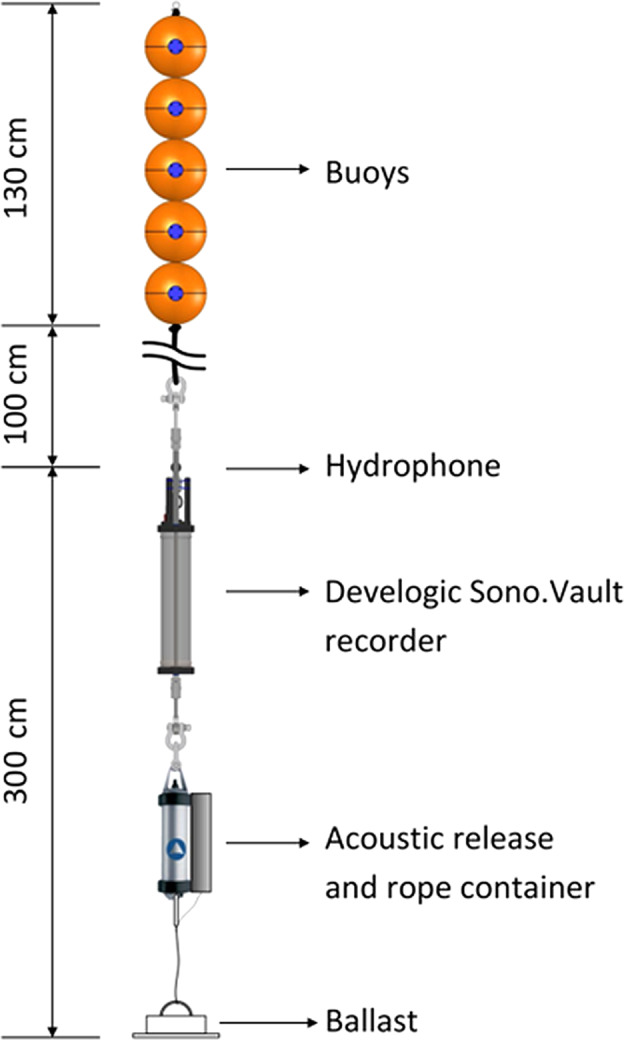
Sketch of the SOUNDSCAPE standard rig deployed on the seafloor, with hydrophone set at ~3 m above the seafloor (range from 2 to 6 m).

A total of nine APUARs were deployed (Fig. 1). In most of the stations, the recorders were anchored to the bottom with a rig design consisting of an anchor, an acoustic releaser, the logger itself secured by polypropylene rope and extra flotations (e.g., sphere with the diameter of 25 cm mounted at minimum of 100 cm from the hydrophone), as illustrated in Fig. 3. The rig design above the anchor was positively buoyant. This ensured that the loggers were suspended about 3 m above the seabed throughout the deployment. In the stations MS1, MS5 and MS6, deployment and recovery operations were carried out by scuba divers, so no acoustic release was needed.

The system calibration was checked *in situ j*ust before the deployment and after the recovery by using an air-pistonphone Grass 42AC (Grass Instruments, West Warwick, RI, USA), that generates a known sound pressure level at 250 Hz. Additionally, profiles of water conductivity, temperature and pressure were recorded by using a CTD probe. Metadata were collected *for* each deployment and recovery, including name, geographic position and the depth of the measurement site, start and stop time for each recording, equipment ID number and set up data, calibration data and weather conditions. Additional details on the deployment and recovery protocols are available in the SOUNDSCAPE Deliverable 3.2.2^43^. Typical measurement duration for the stations was 3 months, after which each device needed to be recovered to download data and to remove biological fouling. The measurement period covered about one full year and four months (from 1 March 2020 to 30 June 2021). Table 3 shows the data coverage *for* each monitoring station.

**Table 3:** Data temporal coverage (days per month) for each recording station.

	MS1	MS2	MS3	MS4	MS5	MS6	MS7	MS8	MS9 D/E
**2020-03-**	1–11	1–31	1–31	1-5/11-31	5–31	1–31		1–31	10–31
**2020-04-**	9–30	1–30	1–22	1–30	1-9/11-30	1–9		1–30	1–30
**2020-05-**	1–31	31		1–31	1–31	7–31	5–31	1–31	1–31
**2020-06-**	1-11/15-30	1–30	10–30	1–30	1-11/14-30	1-11/14-30	1–30	1–30	1–30
**2020-07-**	1–31	1–18	1–31	1–31	1–31	1–31	1–31	1–31	1–22
**2020-08-**	1–31	1–31	1–31	1–31	1-9/13-31	1-9/13-31	1–31	1–31	
**2020-09-**	1–30	1–30	1-10/29-30	1–30	1–30	1–30	1–30	1–29	
**2020-10-**	1–31	1-10/24-31	1–31	1–14	1-18/20-31	1-18/20-31	1–31		
**2020-11-**	1–30	1–30	1–30		1–30	1–30	1–29	25–30	
**2020-12-**	1–31	1–20	1–31		1–31	1–31	4–31	1–31	14-31*
**2021-01-**	1–31	30–31	1–11		1-15/18-31	1-15/18-31	1–31	1–31	1–31
**2021-02-**	1–28	1–28	17–28	5–28	1–28	1–28	1–28	1–28	1–28
**2021-03-**	1-3/11-16	1–31	1–31	1/18-31	1–31	1–31	1–31	1–26	1–31
**2021-04-**		1-3/25-30	1–30	1-4/8-23	1–30	1–30	1–4		1–30
**2021-05-**	4–31	4–31	1-5/14-31	1–10	1–31	1–31	19–31	14–31	1–31
**2021-06-**	1–30	1–30	1–30	17–30	1/4-30	1/4-30	1–30	1–30	1–30

*New position of MS9 since December 2020.

### Acoustic data storage and processing

The collected .wav files were stored on two servers at CNR ISMAR (Venice, Italy) and IOR (Split, Croatia). No data compression was applied to the original files. The whole data-set has been processed by the same processing executable tool, that was developed specially for the SOUNDSCAPE project by the University of Gdansk together with CNR-ISMAR.

The processing steps are briefly summarized:

(i) each 1-sec segment is read from the wav file (i.e. 48000 values, being the Sample Rate equal to 48000 Hz) and a discrete Fourier Transform is applied;
(ii) the power within one third-octave (base 10) band^38^, *U*(*F*), is calculated as
  1 $$U\left(F\right)=\frac{1}{{N}^{2}}{\sum }_{{b}_{1}}^{{b}_{2}}{\left|A\right|}^{2}$$
  where N is the number of samples, A are the coefficients in the discrete Fourier transform and b_1_ and b_2_ are the indices corresponding to the lower and upper frequencies of a given one-third octave band;
(iii) the SPL (L_*p*_) averaged over 1 second (hereafter SPLs_1_ dB re 1 μPa) is obtained as
  2 $${L}_{p}(F)=10{\cdot {\log }}_{10}(U(F))-{S}_{Dev}(F)$$
  where *F* refers to each one third-octave (base 10) frequency band^38^ and *S*_*Dev*_ is a factor related to the Develogic Sono.Vault hydrophone sensitivity, the recording gain and calibration process;
(iv) 20 and 60 seconds averaged SPLs (hereafter SPLs_20_ and SPLs_60_) are then calculated from 1 second averaged SPLs (SPLs_1_) by using the following Eq. (3):
  3 $$SP{L}_{n}=10\cdot \,lo{g}_{10}\left(\frac{1}{n}{\sum }_{i=1}^{n}1{0}^{\frac{SPL{s}_{1i}}{10dB}}\right)dB,$$
  for each SPLs_1i_ with n = 20 or 60;
(v) output data of SPLs_1_, SPLs_20_ and SPLs_60_ are produced.

Specifically, the factor *S*_*Dev*_ in formula (2) is computed by the following formula (4) based on the information provided by the Develogic Sono.Vault manufacturer.

4 $${S}_{Dev}\left(F\right)={S}_{H}\left(F\right)+LUcal+K$$

Where *S*_*H*_ (*F*) (dB/V) is the sensitivity of the hydrophone for each one-third octave frequency band as extracted from the calibration sheet (Table 4), *LUcal* (dB/V) is introduced to take into account the recording gain of the APUAR, *K* is a constant value, being equal to 49.0309 related to the signal used by manufacturer Develogic during the calibration process (see SOUNDSCAPE report^41^ for details).

**Table 4:** Sensitivity (dB/V ref 1 μPa) of the hydrophone S_H_(F) for each one-third octave frequency F extracted from the calibration sheet of the manufacturer Develogic.

F	S_H_(F)
25	−192.70
31	−192.70
40	−192.70
50	−192.70
63	−192.70
80	−192.70
100	−192.70
125	−192.70
160	−192.70
200	−192.70
250	−192.70
315	−192.70
400	−192.70
500	−192.70
630	−192.70
800	−192.70
1000	−192.71
1250	−192.79
1600	−192.90
2000	−192.97
2500	−192.61
3150	−192.36
4000	−192.70
5000	−192.91
6300	−193.44
8000	−194.21
10000	−193.71
12500	−194.43
16000	−195.70
20000	−197.65

## Data Records

The dataset of 20 and 60 seconds averaged Sound Pressure Levels (SPL) output files collected by SOUNDSCAPE and described in this paper is available on Zenodo^44^.

Data are archived using structured HDF5 files, each one containing metadata and SPL data according to the ICES (International Council for the Exploration of the Sea) continuous noise data portal specification, with time stamps relative to UTC time provided in compliance to ISO 8601^45^.

## Technical Validation

In order to ensure the data quality, a check on the collected data by times series visualisation was carried before the data analysis: it did not highlight spurious signals or transient artefacts due to deployment settings, nor systematic artefacts due to flow noise, which is consistent with the study areas being characterized by low tidal currents. Moreover, data recorded before and during the deployment, during and after the retrieval and while the deployment vessel was in close proximity of the recorder were removed.

The measured data may be contaminated by the system self-noise. Self-noise fluctuations in output of an acoustic receiver system are caused by the combination of acoustic self-noise and non-acoustic self-noise (electronic self-noise). The acoustic self-noise sound is usually caused by the deployment, operation, or recovery of a specified receiver and its associated with the deployment of the acoustic sensor and platform (e.g., noise from moorings and fixtures, flow noise, *etc*.) whereas the non-acoustic self-noise is related to fluctuations in the output of a receiver system in absence of any sound pressure input^37^. In the SOUNDSCAPE project, the introduction of unwanted acoustic self-noise in the recordings was prevented by deployment rig’s design and deployment procedure. Attention was given (i) in the mooring settings to minimize the self-noise (i.e., use of soft ropes and avoidance of the metal parts) and (ii) in the positioning of the deployments, by locating them at a distance from the coast that assured no interaction with external infrastructures that could generate unwanted sounds. Further, the monitoring stations were not sited in high tidal flow areas and hydrophones were placed close to the bottom. The SOUNDSCAPE non-acoustic self-noise due to the electrical noise is calculated to be better than 58 dB re 1μPa^2^/Hz at 63 Hz and better than 53 dB re 1 μPa^2^/Hz at 125 Hz, according to the manufacturer technical specifications.

Finally, a quality control has been applied to the software used for the analysis (Fig. 2). To validate the correct functioning of the SOUNDSCAPE processing app (ANP) applied to the wav data, the latter was tested again other already validated software (SpectraPLUS and independent MATLAB routines). A subset of data was processed with the SOUNDSCAPE ANP and the SPLs of each one-third octave (base 10) band were compared with the ones calculated by a validated tool: the SOUNDSCAPE ANP was able to reproduce almost the same results. Namely, the mean absolute difference between trusted tool and SOUNDSCAPE ANP results was equal to 0.08 dB, being less than 0.1 dB in most of the frequencies, with the exception of the lower frequencies (less than 25 Hz), where it was lower than 0.3 dB.

## Usage Notes

To post process the SPLs_20,60_ data, CNR-ISMAR developed a Python script that was deployed as a Jupyter Notebook interactive document^46^, that is here released.

**Fig. 4:**
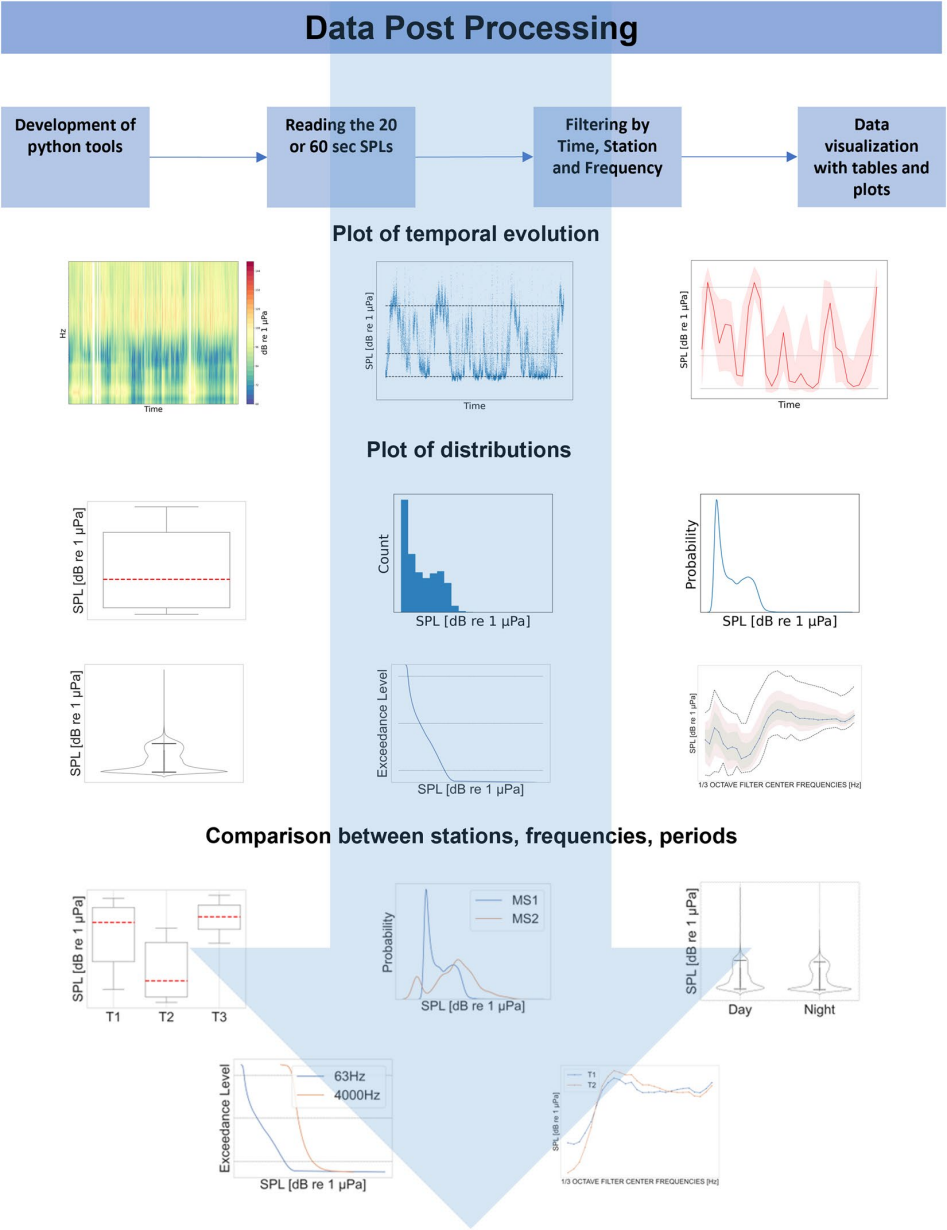
Examples of SPL_20,60_ data post processing outputs generated applying the Python post processing script to the released dataset.

The workflow of SPL data post processing is simple. After reading SPLs_20,60_ files and (i) selecting a time window to define the investigated period, (ii) a recording station and (iii) a given one-third octave (base 10) band, it is possible to compute some metrics to create tables and to visualize efficiently the data (see Fig. 4 for examples). Statistics can be calculated for each one-third octave (base 10) band over various temporal analysis windows (based on UTC time) like for example one hour, one day, one month, one year^10^. Once the time window is selected, tables with descriptive statistics can be produced including percentile values (1^th^, 10^th^, 25^th^, 50^th^, 75^th^, 90^th^, 99^th^percentiles) and the arithmetic mean. The Python script can also generate graphs such as time series plots, to visualize the temporal evolution of SPL data, and descriptive plots, to highlight the principal statistics of the data distribution over the time window. Data can be aggregated also to check their distribution between stations.

## Data Availability

The Jupyter Notebook interactive document for data post-processing is freely available in ROHub, the Research object management platform^47^.
