# Author Correction: First assessment of underwater sound levels in the Northern Adriatic Sea at the basin scale

**DOI:** 10.1038/s41597-023-02099-x

**Published:** 2023-03-30

**Authors:** Antonio Petrizzo, Andrea Barbanti, Giulia Barfucci, Mauro Bastianini, Ilaria Biagiotti, Sofia Bosi, Michele Centurelli, Robert Chavanne, Antonio Codarin, Ilaria Costantini, Marinela Cukrov Car, Vlado Dadić, Francesco M. Falcieri, Raffaela Falkner, Giulio Farella, Mario Felli, Christian Ferrarin, Thomas Folegot, Roger Gallou, Daphnie Galvez, Michol Ghezzo, Aleksandra Kruss, Iole Leonori, Stefano Menegon, Hrvoje Mihanović, Stipe Muslim, Alice Pari, Sauro Pari, Marta Picciulin, Grgur Pleslić, Marko Radulović, Nikolina Rako-Gospić, Davide Sabbatini, Giulia Soldano, Jarosław Tęgowski, Tihana Vučur-Blazinić, Predrag Vukadin, Jakub Zdroik, Fantina Madricardo

**Affiliations:** 1 CNR-National Research Council, ISMAR - Institute of Marine Sciences in Venice, Castello 2737/f, 30122 Venice, Italy; 2 ARPA FVG — Regional Environmental Protection Agency of Friuli Venezia Giulia, via Cairoli 14, 33057 Palmanova, Udine Italy; 3 CNR-National Research Council, IRBIM -Institute of Marine Biological Resources and Biotechnologies, SS Ancona, Largo Fiera della Pesca, 1 - 60125 Ancona, Italy; 4 Quiet Oceans, Bâtiment Cap Ocean, Technopôle Brest-Iroise, 525 avenue Alexis de Rochon, 29280 Plouzané, France; 5 Blue World Institute of Marine Research and Conservation, Kaštel 24, 51551 Veli Lošinj, Croatia; 6 Institute of Oceanography and Fisheries (IOR), Šetalište I. Meštrovića 63, 21000 Split, Croatia; 7 CNR-National Research Council, INM - Institute of Marine Engineering, via di Vallerano 139, 00128 Roma, Italy; 8 NORBIT Poland, ul Niepodleglosci 813-815, lok.24, 81-810 Sopot, Poland; 9 Fondazione Cetacea Onlus, Viale Torino 7A, 47838 Riccione, RN Italy; 10 Institute of Oceanography, University of Gdańsk, Av. Marszałka Piłsudskiego 46, 81-378 Gdynia, Poland

**Keywords:** Environmental sciences, Physical oceanography, Environmental sciences, Physical oceanography

Correction to: *Scientific Data* 10.1038/s41597-023-02033-1, published online 15 March 2023.

In this article the affiliation details for Davide Sabbatini were incorrectly given as ‘NORBIT Poland, ul Niepodleglosci 813-815, lok.24, 81-810, Sopot, Poland’ but should have been ‘Fondazione Cetacea Onlus, Viale Torino 7A, 47838, Riccione, RN, Italy’. The original article has been corrected.

